# Comparison of Tumor Microenvironments Between Primary Tumors and Brain Metastases in Patients With NSCLC

**DOI:** 10.1016/j.jtocrr.2021.100230

**Published:** 2021-09-20

**Authors:** Daiki Ikarashi, Tamio Okimoto, Takehito Shukuya, Hiroko Onagi, Takuo Hayashi, Sara L. Sinicropi-Yao, Joseph M. Amann, Tetsuya Nakatsura, Shigehisa Kitano, David P. Carbone

**Affiliations:** a Division of Cancer Immunotherapy Development, Advanced Medical Development Center, The Cancer Institute Hospital of the Japanese Foundation for Cancer Research (JFCR), Tokyo, Japan; b Division of Cancer Immunotherapy, Exploratory Oncology Research, and Clinical Trial Center, National Cancer Center, Kashiwa, Japan; c Department of Urology, Iwate Medical University, Iwate, Japan; d Division of Medical Oncology, Department of Internal Medicine, James Thoracic Center, The Ohio State University, Columbus, Ohio; e Department of Respiratory Medicine, Juntendo University Graduate School of Medicine, Tokyo, Japan; f Department of Human Pathology, School of Medicine, Juntendo University, Tokyo, Japan

**Keywords:** Non–small cell lung cancer, Primary tumor, Brain metastasis, Microenvironment, Radiation

## Abstract

**Introduction:**

This study investigates the immune profile of the primary lung tumors and the corresponding brain metastasis from patients with NSCLC using multiplex fluorescence immunohistochemistry.

**Methods:**

The study evaluated 34 patients who underwent autopsy or surgical resection for brain metastasis and autopsy, surgical resection, or core biopsy for primary lung cancer. We compared the densities of various immune cells in the primary tumors and the brain metastases by multiplex fluorescence immunohistochemical analysis.

**Results:**

The density of CD4-positive (CD4^+^) T-cells, CD8-positive T-cells, and CD4^+^ Foxp3-positive T-cells were statistically higher in both tumor and stromal areas in primary lung cancer specimens when compared with brain metastases samples (*p* < 0.0001). Only CD204-positive cells were statistically higher in the tumor areas of the brain metastases (*p* = 0.0118). Tumor-infiltrating lymphocytes associated with brain metastases positively correlated with overall survival, but primary lung tumor-infiltrating lymphocytes did not. The density of CD4^+^ and CD4^+^ Foxp3-positive T-cells in brain metastases with radiation was statistically higher in the carcinoma and stromal areas compared with those without radiation (*p* = 0.0343, *p* = 0.0173).

**Conclusions:**

Our findings that CD204-positive cells were higher in brain metastases may have broader implications for treatment as these macrophages may be immunosuppressive and make the immune environment less reactive. Furthermore, the finding that the density of CD4^+^ T-cells was higher in cancer and stroma areas of brain metastases after radiotherapy supports the addition of immunotherapy to radiation therapy in the treatment of brain metastases in NSCLC.

## Introduction

Brain metastasis is a major cause of lung cancer death.^1^ Historically, the median survival of patients with lung cancer with untreated brain metastasis is only 1 to 2 months after diagnosis of their brain metastases.^2^ New treatment modalities for brain metastasis including stereotactic radiosurgery have led to better outcomes, but there is a tremendous need for improvement. Because available therapies have the risk of neurologic worsening, intracranial hemorrhage, seizures, and cognitive impairment, repetitive use of these treatments needs to be carefully considered. The efficacy of systemic therapy with conventional cytotoxic agents for brain metastasis is limited owing to the blood-brain barrier. In multiple clinical trials, the efficacy of immunotherapy in treating brain metastases has been reported; however, it is a frequent site of the first relapse after immunotherapy.^3^

The development of cancer immunotherapy dramatically changed the treatment strategy for NSCLC. An immunotherapy-containing regimen is now recommended as first-line therapy for those patients without an actionable driver mutation.^4^^,^^5^ Although programmed death-ligand 1 (PD-L1) expression on tumor cells is the most established biomarker to predict the efficacy of anti–programmed cell death protein 1 or anti-PD-L1 therapy for NSCLC,^6^, ^7^, ^8^, ^9^ the heterogeneity of PD-L1 expression has been reported between the primary lung tumors and metastases.^10^^,^^11^

The tumor microenvironment (TME) also plays an essential role in response to immunotherapy.^12^, ^13^, ^14^, ^15^ A high density of tumor-infiltrating lymphocytes (TILs) and an inflamed tumor state are associated with greater immune responsiveness. Despite the fact that a reactive TME, including PD-L1 expression, is key to successful immunotherapy, it is unknown whether the TME in the primary site is representative of that in brain metastases.

TME can be also be affected by treatment, as radiation therapy and chemotherapy may be able to induce immunogenic cell death depending on the type of regimen and method of intervention. Tumor antigens derived from dead and dying tumor cells are processed by dendritic cells and presented by major histocompatibility complex class I and II molecules to antigen-specific CD8-positive (CD8^+^) and CD4-positive (CD4^+^) T-cells, respectively, which leads to immune activation.^16^ Moreover, even if these treatments are not cytotoxic, they make the tumor cells undergo irreversible proliferative arrest. These cells can display a senescence-associated secretory phenotype, in which they secrete proinflammatory molecules and enhance antitumor immunity.^16^, ^17^, ^18^, ^19^ Local treatment for brain metastasis is mainly performed with radiation therapy, but the effect of the treatment on TME remains largely unexplored.

The infiltration of the immune cells into the TME can be a prognostic factor of NSCLC.^20^, ^21^, ^22^, ^23^ Reports have found that a higher number of CD4^+^ T-cells and CD8^+^ T-cells in the stroma, but not in the areas of cancer cells, is associated with a better prognosis after surgery for NSCLC.^20^ These studies also indicate that the spatial distribution of the immune cells in the tumor is important. However, conventional techniques used in many tumor analyses, such as flow cytometry, and sequencing analysis, cannot distinguish the localization of various types of immune cells in the tumor.

To shed light on the immune environment in primary lung tumors and associated brain metastases (and definitively determine immune cell localization), we investigated the immune profile of primary tumors and the corresponding brain metastases from 34 patients with NSCLC with multiplex fluorescence immunohistochemistry (mFIHC). This technology enabled us to assess the immune profiles of the areas of the cancer cells and the stroma separately. We determined that the brain metastases, carcinoma, and stromal areas, contained fewer immune cells than the paired primary lung cancer, except for CD204-positive (CD204^+^) cells, which were higher in the carcinoma areas of the brain metastases. In addition, a higher number of CD4^+^ T-cells was observed in carcinoma and stromal areas of brain metastases after local radiotherapy to the brain, which was associated with longer overall survival (OS). To the best of our knowledge, this is the first report illustrating differences in the immune environment of primary lung tumors and paired brain metastases, before and after radiation therapy, in patients with NSCLC.

## Materials and Methods

### Patient Data

We retrospectively evaluated 34 patients who underwent autopsy or surgical resection for brain metastasis and autopsy, surgical resection, or core biopsy for primary lung cancer at The Ohio State University (OSU) between January 1, 1988 and June 28, 2018. Several clinical factors, including age, sex, smoking history, and postmedical history, were recorded. Archival samples were obtained from the OSU Tissue Archive Services or the Total Cancer Care Biorepository through a waiver-of-consent process. This study was approved by the OSU Institutional Review Board (approval number: 2015C0046) and was conducted in accordance with the Declaration of Helsinki provisions.

### Multiplex Immunofluorescence Assay and Analysis

The 4-μm thickness tissue sections obtained from formalin-fixed paraffin-embedded tissue blocks were subjected to mFIHC staining using the Opal Kit (AKOYA Biosciences, Marlborough, MA). The antibodies, dilutions, and activation conditions used are listed in Supplementary Table 1. The slides were scanned using the Vectra slide scanner (AKOYA Biosciences). For each marker, the mean fluorescent intensity per case was then determined as a base point from which positive calls could be established. For multispectral analysis, each individually stained section was used to establish the spectral library of the fluorophores. Five to 20 random areas on each sample were analyzed blindly by two pathologists at ×20 magnification.

The summary for analysis is described below, following previously reported methods.^24^, ^25^, ^26^ An image analysis program (Inform; AKOYA Biosciences) was used to segment tumor tissues into carcinoma and stromal areas and to detect immune cells with specific phenotypes; after which, the distribution of immune cells was analyzed. Training sessions for tissue segmentation and phenotype recognition were carried out repeatedly until the algorithm reached the level of confidence recommended by the program supplier (at least 90% accuracy). After phenotyping typical CD4^+^ and CD8^+^ cells using the Inform software, gated CD3-positive (CD3^+^) populations by mean fluorescence intensity of CD3, CD3^+^CD4^+^, and CD3^+^CD8^+^ cells were determined as CD4^+^ T-cells and CD8^+^ T-cells, respectively. A similar gating strategy was used for the analysis of Foxp3-high population in CD4^+^ T-cells and PD-L1–high population in cancer cells using an analytical program (Spotfire version 7.8; TIBCO Software, Palo Alto, CA). The area of each tissue category, divided into carcinoma and stroma, was evaluated to assess the density of each immune cell type, represented as follows: density of immune cells = counts of immune cells/areas of cancer and stromal (mm^2^). T-cells in the carcinoma and stromal areas were defined as carcinoma T-cells and stromal T-cells, respectively.

### Statistical Analysis

Wilcoxon signed rank tests were done when comparing values of the densities of immune cells between lung primary lesions and brain metastases. The statistical comparison between the untreated brain metastasis group and the brain metastasis group receiving radiation before surgery for a given continuous variable was performed using Mann–Whitney *U* test. OS was measured from the date of brain surgery to death in all cases. A linear regression model was performed among densities of each immune cell and OS. All *p* values were two-sided, and *p* values of less than 0.05 denoted statistical significance. All statistical analyses were performed using the JMP software (SAS Institute, Inc., Cary, NC).

## Results

Patient characteristics are described in Table 1. The median age was 60.5 years (range: 36–77 y), and 50% of patients were men. Patients were mostly white (88%). A total of 15 patients had adenocarcinoma (44%) histologic diagnosis—seven patients had an adenosquamous cell carcinoma (21%), three squamous (9%), five large cell (15%), and three being not otherwise specified (9%). The median interval between the lung surgery and the brain metastasectomy was 335.5 (range: 0–2618) days.

**Table 1:** Patient Characteristics

Characteristic	N = 34
Sex	
Male	17
Female	17
Age (y)	
Median	60.5
Range	36–77
Race	
White	30
Black	1
Hispanic	1
Unknown	2
Histotype	
Adenocarcinoma	15
Squamous cell carcinoma	3
Adenosquamous cell carcinoma	7
Large cell carcinoma	5
Not otherwise specified	3
EGFR mutation	
Positive	3
Negative	17
Unknown	14
ALK fusion	
Positive	2
Negative	12
Unknown	20
Smoking status	
Current	12
Former	18
Never	1
Unknown	3
Interval between lung and brain tumor sampling (d)	
Median	335.5
Range	0–2618
Clinical stage at the time of lung tumor sampling	
Nonmetastatic	23
Metastatic	9
Unknown	2

**Figure 1:**
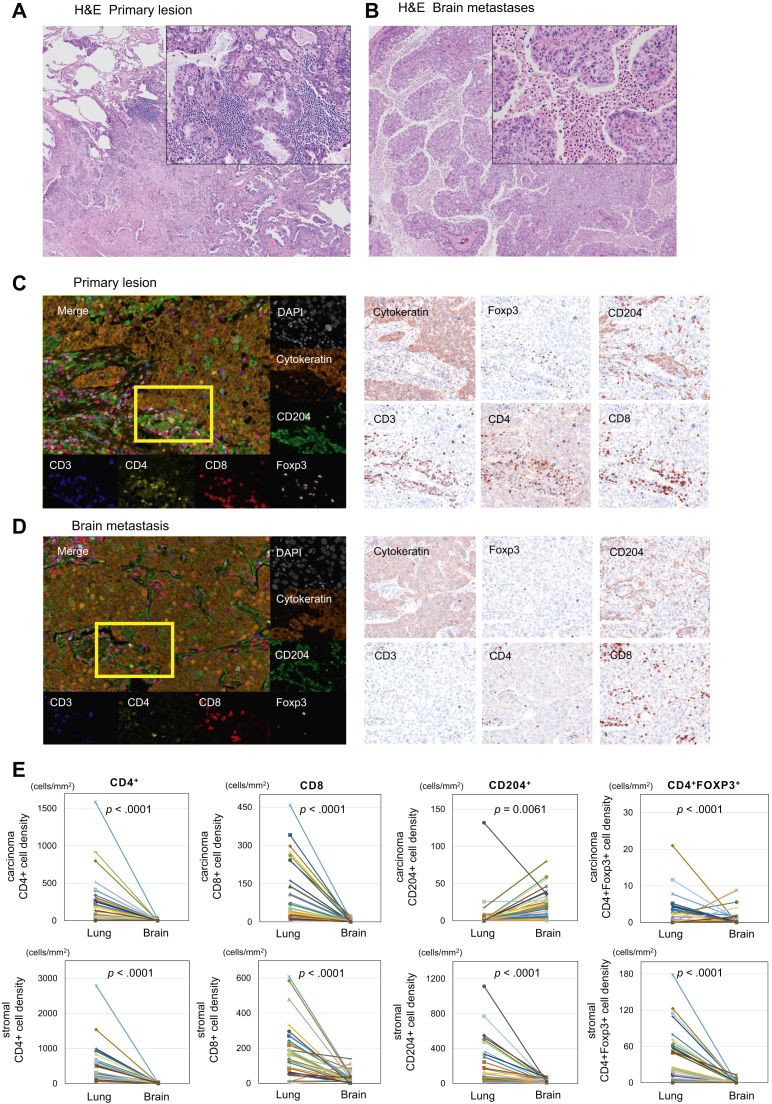
Representative image of H&E staining in (*A*) lung cancer (×40, ×200) and (*B*) brain metastasis specimens (×40, ×200). Multiplex immunofluorescence in (*C*) lung cancer and (*D*) brain metastasis specimens for the following markers: CD3, CD4, CD8, CD204, Foxp3, cytokeratin, and DAPI. Original magnification, ×20. Additional staining in (*C*)**,** (*D*) illustrating associated singlet immunostaining of CD3, CD4, CD8, CD204, Foxp3, and cytokeratin. (*E*) Relationship of the density of each immune cell in the cancer area and the stromal area between lung cancer and brain metastasis specimens. CD204^+^, CD204-positive; CD4^+^, CD4-positive; CD8^+^, CD8-positive; DAPI, 4′,6-diamidino-2-phenylindole fluorescent stain; Foxp3^+^, Foxp3-positive; H&E, hematoxylin and eosin staining.

**Figure 2:**
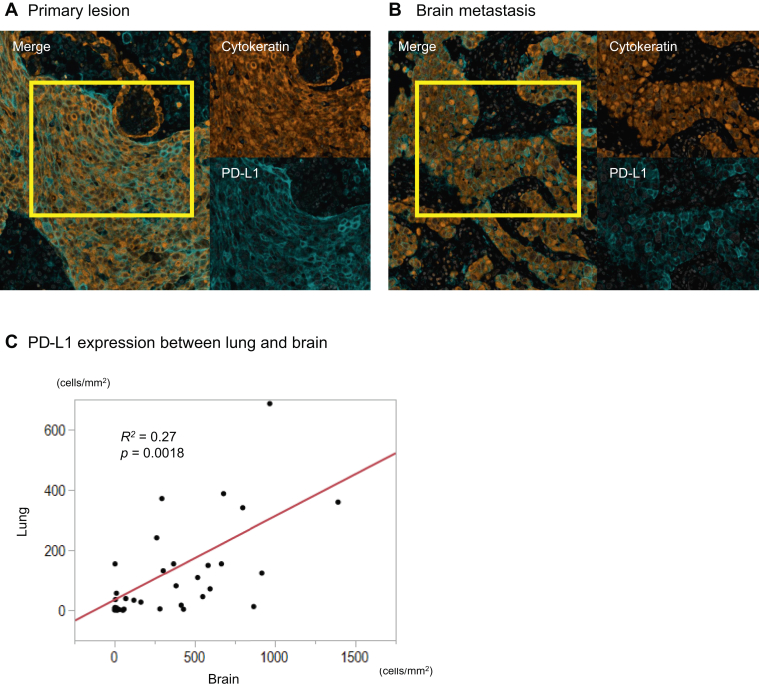
Multiplex immunohistochemistry images of PD-L1 expression on cancer cells among (*A*) lung cancer and (*B*) brain metastasis specimens. (*C*) Relationship of PD-L1 among (*A*) and (*B*). Correlation plots of PD-L1 expression on cancer cells between lung cancer and brain metastasis across all samples. Scores were significantly correlated (*p* = 0.0018). PD-L1, programmed death-ligand 1.

We assessed the numbers of immune cells in primary lesions and brain metastases by hematoxylin and eosin staining and mFIHC (Fig. 1*A–D*). The density of CD4^+^ T-cells, CD8^+^ T-cells, and CD4^+^ Foxp3-positive (Foxp3^+^) T-cells were statistically higher in both carcinoma and stromal areas in the primary specimens when compared with matched brain metastases (*p* < 0.0001, respectively). Only CD204^+^ cells were statistically higher in the carcinoma areas in brain metastases when compared with matched primary lung cancer (Fig. 1*E*) (*p* = 0.0061). We also assessed PD-L1 expression on cancer cells between the primary lesions and brain metastases (Fig. 2*A–B*) and found that PD-L1 expression was significantly correlated (Fig. 2*C*) (*p* = 0.0018). These results reveal that primary lung lesions have more immune cells than the paired brain metastases, except CD204^+^ cells, which are higher in the carcinoma areas of the brain metastases.

**Figure 3:**
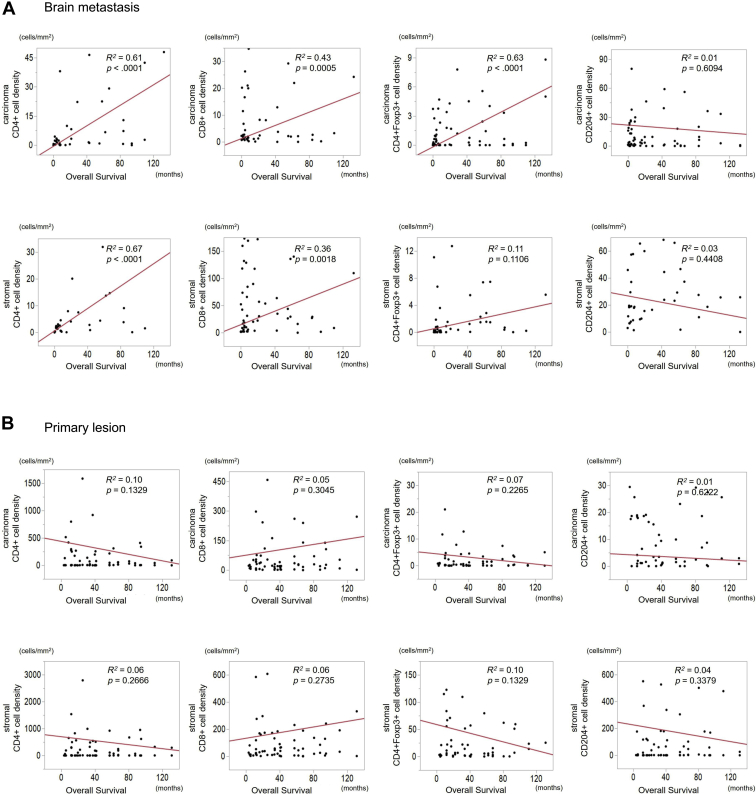
Linear regression modeling of the density of CD4^+^ T-cell, CD8^+^ T-cell, CD204^+^ cell, and CD4^＋^Foxp3^＋^ T-cell in cancer and stromal area values for overall survival as indicated. (*A*) brain metastases (*B*) primary lesion. CD4^+^, CD4-positive; CD8^+^, CD8-positive; CD204^+^, CD204-positive; Foxp3^+^, Foxp3-positive.

To determine whether these differences in the immune cell profiles had any relationship with the outcome, we evaluated the correlation of immune cell density in the carcinoma and stromal areas with OS. The densities of CD4^+^ T-cells and CD8^+^ T-cells in the carcinoma and the stromal areas of the brain metastases were positively correlated with OS by linear regression models (Fig. 3*A*). The densities of CD4^+^Foxp3^+^ cells were positively correlated with OS in the carcinoma area of the brain metastases, but not in the stromal areas. The densities of CD204^+^ cells in the carcinoma and the stromal areas of the brain metastases did not correlate with OS. With regard to the primary lesions, the densities of CD4^+^ T-cells, CD8^+^ T-cells, CD204^+^ cells, and CD4^+^Foxp3^+^ T-cells in carcinoma and stromal areas were not statistically correlated with OS by linear regression models (Fig. 3*B*). Here we have found that primary lung cancer TILs do not correlate with OS, whereas for brain metastases, TILs do correlate, despite their lower numbers.

**Figure 4:**
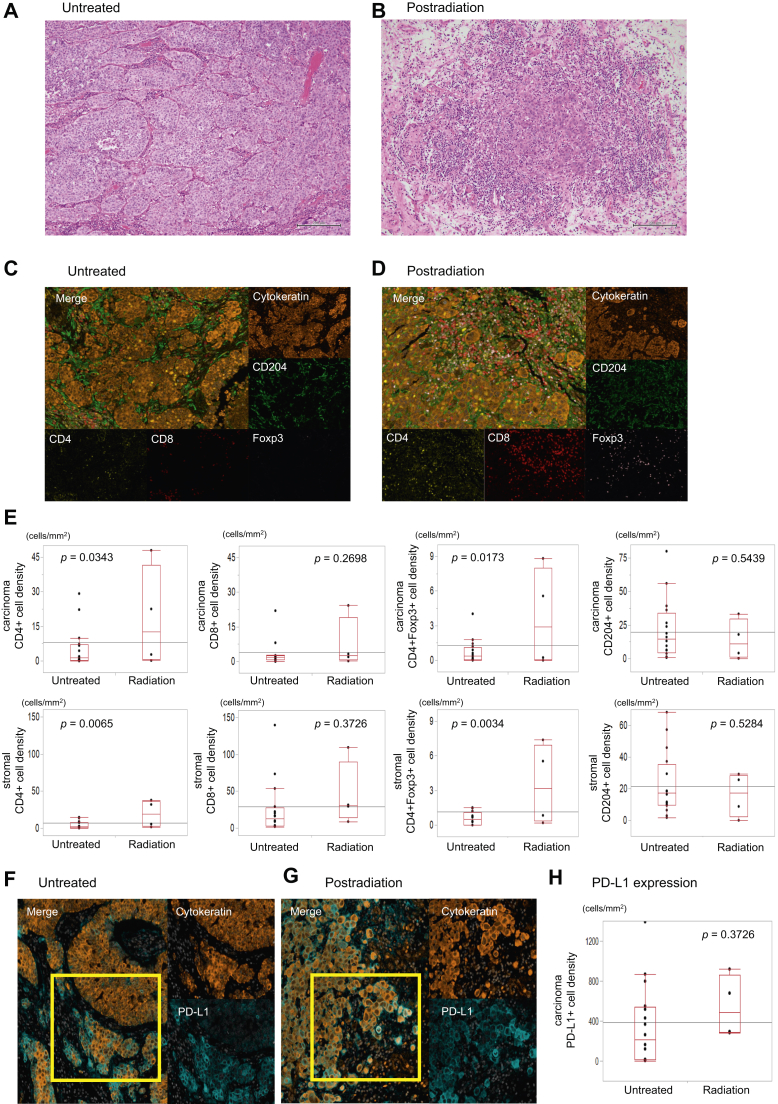
(*A–D*) H&E staining (×100) and multiplex immunohistochemistry images from untreated brain metastasis specimens (n = 19) and treated with radiation specimens (n = 3). Staining as indicated and similar to Fig. 1. (*E*) Relationship of the density of each immune cell in the cancer area and the stromal area between untreated and postradiation brain metastasis specimens. (*F, G*) Multiplex immunohistochemistry images of PD-L1 expression on cancer cells among them. (*H*) Relationship of the density of PD-L1 expression on cancer cells between untreated and postradiation brain metastasis specimens. CD204^+^, CD204-positive; CD4^+^, CD4-positive; CD8^+^, CD8-positive; Foxp3^+^, Foxp3-positive; H&E, hematoxylin and eosin staining; PD-L1, programmed death-ligand 1.

To evaluate the effects of radiotherapy on the immune profile in brain metastases, we assessed the immune cells and PD-L1 expression on brain metastases samples with or without a history of radiation therapy. The patients were divided into a postradiation group (n = 4) and an untreated group (n = 18)—that is, no radiation or chemotherapy before brain surgery (Table 2). Of the patients that received radiotherapy, three received whole-brain radiotherapy, and one received stereotactic radiation therapy. The median interval between brain radiotherapy and brain surgery was 109 (range: 6–390) days. We excluded patients treated with chemotherapy before brain surgery. We observed a higher number of lymphocytes in the hematoxylin and eosin–stained brain specimens in the postradiation group compared with the untreated group (Fig. 4*A–B*). The densities of CD4^+^ and CD4^+^Foxp3^+^ T-cells in the radiation group were statistically higher than the untreated group in carcinoma and stromal areas (*p* = 0.0343, *p* = 0.0173) (Fig. 4*C–E*). The densities of CD8^+^ T-cells and CD204^+^ cells were not statistically significant between the postradiation group and the untreated group (*p* = 0.2698, *p* = 0.5439). We also evaluated the difference in PD-L1 expression on carcinoma cells between these two groups and found that it was not statistically significant (*p* = 0.3726) (Fig. 4*F–H*).

**Table 2:** Patient Characteristics With or Without Brain Radiotherapy

Characteristic	Brain Radiotherapy− (n = 18)	Brain Radiotherapy+ (n = 4)
Sex		
Male	7	4
Female	11	0
Age (y)		
Median	66	57
Range	44–74	36–61
Race		
White	16	4
Black	0	0
Hispanic	1	0
Unknown	1	0
Histotype		
Adenocarcinoma	8	1
Squamous cell carcinoma	3	0
Adenosquamous cell carcinoma	2	1
Large cell carcinoma	3	2
Not otherwise specified	2	0
EGFR mutation		
Positive	1	0
Negative	10	2
Unknown	7	2
ALK fusion		
Positive	2	0
Negative	6	1
Unknown	10	3
Smoking status		
Current	4	2
Former	11	1
Never	1	0
Unknown	2	1
Interval between lung and brain tumor sampling (d)		
Median	255	287
Range	0–2234	42–668
Clinical stage at the time of lung tumor sampling		
Nonmetastatic	11	1
Metastatic	6	2
Unknown	1	1

## Discussion

In this study, we have revealed that brain metastases have fewer TILs, such as CD4^+^ T-cells and CD8^+^ T-cells, when compared with the primary lesion from the same patient. Only CD204^+^ cells were statistically higher in the carcinoma areas in brain metastases, but not in the stromal areas. A previous study in NSCLC reported similar results that compared the immune microenvironments of primary and metastatic breast tumors and observed relatively fewer TILs in brain metastases compared with primary sites by immunohistochemistry (IHC).^27^^,^^28^ The prognostic value of carcinoma versus stromal lymphocytic infiltration has been reported in NSCLC, with only high density CD4^+^/CD8^+^ stromal lymphocyte infiltration being an independent positive prognostic indicator for patients with resected NSCLC.^20^

Most previous studies have assessed the local cancer microenvironment by standard IHC. However, with standard IHC, it has been difficult to analyze the interrelation of stained immune cells, cancer cells, and stroma. Furthermore, some researchers believe that distinguishing between carcinoma and stromal immune infiltration is difficult to assess objectively because of the lack of interobserver reproducibility.^23^ We have assessed immune cells in tumors by mFIHC, which is a useful tool for comprehensive analysis of immune cell type and allows for easy identification of regions in which specific immune cells are located in the cancer microenvironment.^29^^,^^30^ In this study, we used an image analysis program (Inform), which enabled us to distinguish immune cells and their location more accurately and objectively. For example, whereas the density of CD4^+^Foxp3^+^ T-cells in the carcinoma areas exhibited a positive correlation with survival after brain tumor resection, their density in stromal areas did not. Therefore, we believe that spatial analysis of TILs leads to a better understanding of the TME and how it might affect the outcome. To our knowledge, this is the first study to report the use mFIHC to visualize the microenvironment of brain metastases and compare them to primary lesions from the same patient.

In this study, we have illustrated that the densities of CD4^+^ T-cells and CD8^+^ T-cells in carcinoma and stromal areas, and CD4^+^Foxp3^+^ T-cells in the carcinoma area of the brain metastases were positively correlated with OS. Interestingly, the immune cells located in carcinoma and stromal areas of the primary lung cancer did not correlate with OS, which is in contrast with a previous report in which stated that primary lung cancer TILs in stromal areas do correlate with OS.^20^ However, contrary to the previous report, all patients in our study had brain metastases. It is likely that patients with lung cancer and brain metastases are at a higher risk of mortality than those with resected primary lung cancer without brain metastases. Thus, despite low numbers of TILs in the brain metastases, this may explain why, in our study, TILs in brain metastases correlate with OS and TILs in primary lung cancers do not. In addition, we observed no correlation of TILs in the primary lesion and progression-free survival (Supplementary Fig. 1). The reason for this may be owing to the difference in cancer stage at the time of collecting the specimen and subsequent treatment.

It seems paradoxical that we saw a positive correlation between OS and the densities of CD4^+^Foxp3^+^ T-cells in tumors because many reports in NSCLC indicate that regulatory T-cell (Treg) infiltration is a poor prognostic factor.^31^^,^^32^ However, it has also been reported that infiltration of CD4^+^Foxp3^+^ T-cells is associated with a favorable prognosis in some types of cancers.^32^^,^^33^ Perhaps the increase in CD4^+^ Tregs reflects a reaction to the adaptive immune response. When compared with gliomas, which seem to be infiltrated predominantly by tumor-associated macrophages (TAMs), brain metastases from other cancer types (melanoma brain metastases, in particular) are infiltrated largely by CD4^+^ and CD8^+^ T-cells.^34^, ^35^, ^36^ Many studies have reported that expression of Foxp3 not only occurs in Tregs, but also occurs weakly in activated T-cells and it would be difficult to distinguish these subpopulations by standard IHC.^37^ In addition, Tregs migrate mainly into the sites of inflammation and inhibit various types of lymphocytes, such as CD4^+^ helper T-cell and CD8^+^ cytotoxic T lymphocytes.^38^ Consequently, the high density of CD4^+^Foxp3^+^ T-cells in the tumor immune microenvironment could represent a state of immune activation, which may be the reason for the correlation between the density of CD4^+^Foxp3^+^ T-cells and OS in this study. In addition, we saw that the densities of CD204^+^ cells in the primary tumor and the brain metastases did not correlate with OS. Although it has been reported that TAMs can be a poor prognostic factor in many types of tumors, in a recent review, most studies stated that CD204^+^ cells are not always a prognostic factor of poor outcome in lung cancer.^34^^,^^39^

Radiation therapy is reported to be a powerful tool to modulate the local immunologic properties of the tumor and to promote an antitumor response.^40^^,^^41^ Localized radiation initiates cell death and the production and release of cytokines and chemokines into the TME by means of type Ⅰ interferons. This leads to infiltration of cytotoxic T-cells and suppressive cells, such as Treg cells, and the efflux of immune cells, such as dendritic cells that are important antigen-presenting cells.^42^ With regard to how radiation affects the immune environment of brain metastases, we evaluated brain metastases from a small number of patients that received cranial radiation before surgery and compared them with metastases from patients that did not receive radiation. For those patients who experienced local radiation to their brain metastases before brain surgery, we observed more TILs localized to the metastases than metastases from patients that did not receive radiation before brain surgery. This suggests that local radiation for brain metastases before surgery might improve prognosis by promoting immune activation. In fact, the density of CD4^+^ and CD4^+^Foxp3^+^ T-cells in the radiation group was statistically higher than the untreated group in both areas of the carcinoma and the stroma. Berghoff et al.^43^ reported that TILs were common in brain metastases of multiple cancer types and were positively correlated with prognosis. In addition, the number of CD8^+^ T-cells in the radiation group tended to be higher than the untreated group, although this was not statistically significant (*p* = 0.2698).

Our data may have implications for the effectiveness of combined immune checkpoint inhibitor and radiotherapy. It is well known that TILs play a role in the efficacy of immune checkpoint inhibitor therapy and increasing the number of TILs through radiation could be a way to increase the effectiveness of immunotherapy for brain metastases. Indeed, some retrospective studies have suggested the efficacy of combination therapy.^44^, ^45^, ^46^, ^47^ A prospective clinical trial is needed for advanced NSCLC with brain metastasis.

To best of our knowledge, this is the first study to analyze the immune microenvironment between the primary lesion and the paired brain metastasis in lung cancer by mFIHC. Limitations include that this is a relatively small study and the fact that resections were not synchronous so that the impact of the interval between lung and brain resections on the TME in brain metastases versus primaries cannot be assessed. However, it does reveal the use of this methodology for the analysis of primary tumors and associated brain metastases. The associated software leads to improved accuracy and objectivity and provides a spatial reference for the immune cells within the cancer cells and the stroma. Additional retrospective and prospective studies with larger sample sizes and comparisons of other solid tumors are needed to validate our results. Our study is limited in the ability to distinguish CD204^+^ cells and microglia in the brain TMEs. However, a previous study assessed the expression of TMEM119, a microglia marker, to distinguish between the two using IHC, because gene expression profiling does not allow accurate discrimination between peripherally derived macrophages and brain resident microglia. This analysis revealed that most of CD68^+^ immune cells in the brain metastases were negative for TMEM119, and thus, represented macrophages.^27^ Moreover, Komohara et al.^48^ reported that the ratio of M2 macrophages in the TAMs/microglia was associated with the histologic grade. Therefore, we believe CD204^+^ microglia will not substantially affect our clinical conclusions.

In conclusion, our results revealed that in the TME in brain metastases there were fewer immune cells when compared with paired primary lesions, except for CD204^+^ cells. However, despite their lower numbers, TILs in brain metastases were positively correlated with OS. In addition, we found that the patients who experienced local radiation to the brain metastases before brain surgery observed a higher number of CD4^+^ T-cells in cancer areas and stroma of brain metastases. We speculate that these higher TIL numbers postradiation may indicate immune activation. We believe that our findings can contribute toward further understanding intratumoral immune cell behavior and a hypothesis that provides a rationale for the combination of immunotherapy and radiotherapy for the treatment of brain metastases.

## CRediT Authorship Contribution Statement

**Daiki Ikarashi**: Methodology, Formal analysis, Investigation, Writing - original draft, Visualization.

**Tamio Okimoto**: Formal analysis, Investigation, Writing - original draft, Visualization.

**Takehito Shukuya**: Conceptualization, Supervision.

**Hiroko Onagi**, **Takuo Hayashi**: Visualization.

**Sara L. Sinicropi-Yao**: Conceptualization.

**Joseph M. Amann**: Resources, Writing - review & editing.

**Tetsuya Nakatsura**: Methodology, Resources.

**Shigehisa Kitano**: Resources, Visualization, Supervision, Project administration, Funding acquisition.

**David P. Carbone**: Resources, Supervision, Project administration, Funding acquisition.
