# Supplementary material for: Comparison of Tumor Microenvironments Between Primary Tumors and Brain Metastases in Patients With NSCLC
Source: JTO Clin Res Rep. 2021 Sep 20;2(10):100230. doi: 10.1016/j.jtocrr.2021.100230 (PMC8501504; doi:10.1016/j.jtocrr.2021.100230)
Supplement: supplemental figure 1 [file mmc1.pptx]

## Slide 1
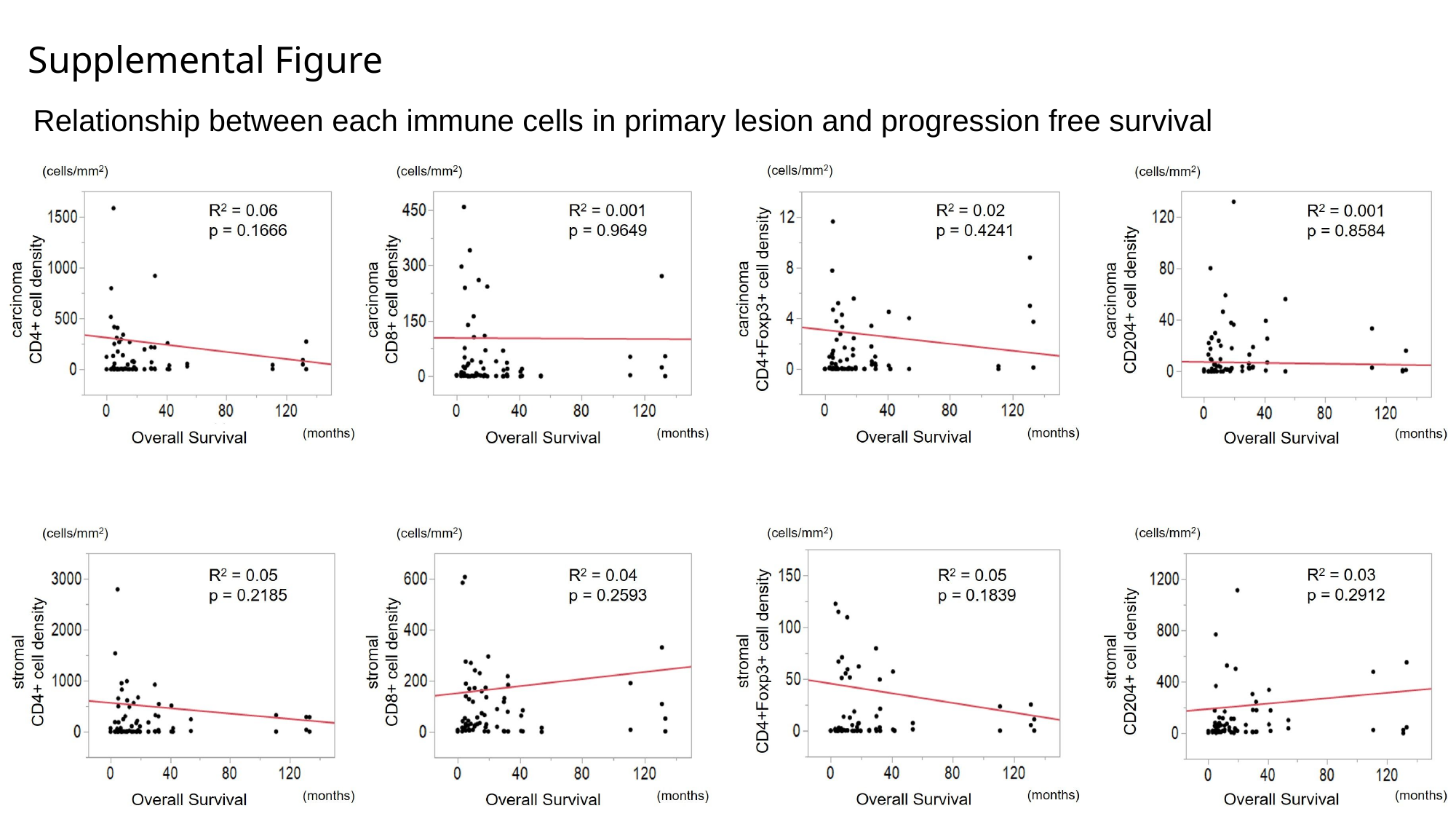

Supplemental Figure
Relationship between each immune cells in primary lesion and progression free survival
